# Research and Remedies

**Published:** 1912-04-06

**Authors:** 


### RESEARCH AND REMEDIES.

### Tuberculin + Sanatorium.

A brief paper contributed to the *Lancet* by Dr. Radcliffe, pathologist to the King Edward VII. Sanatorium at Midhurst, closes with an interesting summary of the author's opinions on the relative virtues of the sanatorium and of tuberculin in the treatment of consumption. With sanatorium treatment alone he reckons on 20 to 25 per cent. of patients ceasing to expectorate bacilli as an immediate result of treatment. When tuberculin is combined with sanatorium treatment the percentage rises to 50. A comparison of the immediate results, he argues, is so much in favour of tuberculin that it is difficult to understand the opposition to it, both in diagnosis and treatment. This view is interesting because at Midhurst tuberculin treatment has not been adopted. Dr. Radcliffe's statistics in support of his contentions are got by comparing the results obtained at Midhurst with those from certain German sanatoria at which the combined treatment is in vogue. It is hardly necessary to point out that there may be other factors explicatory of the differences, such as the way in which the patients are selected.

### The Growth of Bone.

The current teaching of the surgical and physiological text-books concerning the growth of bone is that which has been for many years handed on as it was received from a former generation. Medical students—the surgeons and physicians of to-morrow—are taught in good faith on the strength of experiments of ancient date that new bone can be formed by the periosteum, the strong fibrous adherent sheath in which all bones are encased; and that during natural growth in young animals the whole of the increase in length is due to formation of new bone at two or more points named epiphyses, which in the limbs are situated quite close to the ends of the bones. When a bone is fractured the regeneration of its shaft is believed to be due to the bone-forming activity of the periosteum, and when the periosteum is extensively detached from the bone, or destroyed, it is held that new bone will not then be formed.

These views Sir William Macewen* does not subscribe to. He believes that the periosteum has no osteogenetic (bone-forming) power at all. As a limiting and protecting membrane it is of great use, but it can neither produce nor reproduce bone. Indeed he thinks one of its functions is to limit the growth of bone and prevent the bone-forming cells from extending their activities into the soft tissues. He also holds that the bone-forming cells are not confined to the epiphyses; but that new bone can also be produced in the diaphyseal part of the shaft, quite independently of the periosteum. The growth in length of bones is mainly carried out in the epiphyses; that, of course, no surgeon can possibly question. Yet Sir W. Macewen finds that if these are artificially removed some growth takes place from the diaphyses. For the details of the experiments upon animals and observations upon patients which have led him to these conclusions, the book itself must be consulted. The applications of these researches to surgery are, naturally, very important, and Sir William's teaching is calculated to modify very considerably current surgical practice in many directions. Thus he has shown by operations upon human subjects that osseous grafts to repair deficiencies in the bones of the skull, limbs, etc., can be quite successfully used, even although there is no periosteum attached to them. A number of skiagrams from actual cases support the clinical evidence cited. In one case a patient in whom a large part of the lower jaw had been destroyed had the gap filled by a piece cut from one of her own ribs. Not only did the graft "take," and so remedy a most horrible deformity, but the gap in the rib soon filled up. We are so much accustomed to look to the Continent for all pioneer work in orthopædic surgery, that these valuable contributions to science by a British surgeon are doubly welcome.

* *The Growth of Bone*, by Sir Wm. Macewen, M.D., F.R.S. Glasgow: James Maclehose and Sons. Price 10s. net.

### Fatty Infiltration v. Fatty Degeneration.

For some time the old views on the pathology of fatty changes have been undergoing modification, as Sir Clifford Allbutt stated in a recent address. In the light of various researches it has been held that there is no such process as fatty degeneration, as distinct from fatty infiltration. Although all pathologists do not subscribe to this view, it is interesting to note that some recent work in America tends to confirm those who first propounded it. Dr. F. M. Hanes publishes in the *Johns Hopkins Hospital Bulletin* a highly technical paper on the chemistry of fat metabolism, and his views are substantially as follows: Besides the neutral fats, fatty acids, and soaps, there are a number of more complex substances which are of great practical importance in the study of the normal and abnormal metabolism of fats. The whole group, simple and complex, he terms lipins; they are constant-cell constituents, but are usually invisible in the healthy cell. Fatty accumulation in a cell is not the result of a degenerative transformation of the cytoplasm into fat, but is simply a deposit of lipins which, owing to some injury, the cell cannot utilise. Such deposits may be physiological or pathological, but are always an infiltration, not a degeneration. Pathological fatty infiltrations are of three varieties: glyceryl-ester infiltrations, cholesteryl-ester infiltrations, and infiltrations of other lipins. The first of these indicates an injury to the containing cell, the others are evidence of necrobiosis in neighbouring cells. Such lines of chemico-physiological research as are here opened up may, and probably will, lead eventually to the solution of many problems of life and death which are at present obscure.

